# Abrin Toxin Paradoxically Increases Protein Synthesis in Stimulated CD4^+^ T-Cells While Decreasing Protein Synthesis in Kidney Cells

**DOI:** 10.3390/cimb46120835

**Published:** 2024-12-11

**Authors:** Bradley Hernlem, Reuven Rasooly

**Affiliations:** Foodborne Toxin Detection and Prevention Research Unit, Western Regional Research Center, Agricultural Research Service, United States Department of Agriculture, Albany, CA 94710, USA; bradley.hernlem@usda.gov

**Keywords:** abrin, T-cells, cytokines, autoimmune demyelinating disease, superantigen

## Abstract

Abrin, a toxin of the rosary pea plant (*Abras precatorius*), has been implicated as causing an autoimmune demyelinating disease in humans, but the exact mechanisms responsible for the induction of these demyelinating conditions are still unknown. Certain superantigen microbial toxins such as Staphylococcus enterotoxin type A, type D, type E or streptococcal pyrogenic exotoxin type C also lead to various diseases including autoimmune disorders of the nervous system. Here, the effect of abrin toxin on the immune reaction was studied in human CD4^+^ T-cell lines, and its inhibition of protein synthesis in kidney cells. It is shown for the first time that low concentrations of abrin toxin up to as high as 1 to 10 ng/mL amplifies superantigen activity in stimulated T-cells, leading to excessive NFAT pathway activation and secretion of cytokines, e.g., interleukin-2 (IL-2) and interferon-γ (INFγ), in a dose-dependent manner. This behavior, except at high concentration, is contrary to the effect on other cell types. Abrin’s inhibition of protein synthesis was demonstrated with Vero (kidney) cells and milk was observed to competitively reduce this effect. This new concept in the behavior of abrin in amplifying superantigen activity may explain the mechanism by which abrin toxin triggers autoimmune demyelinating disease in people exposed to low doses of the toxin via the excessive secretion of cytokines which may create excessive inflammation leading to loss of immune tolerance and triggering an immune response against self-antigens.

## 1. Introduction

Abrin, a proteinaceous toxin found in the seeds of the plant *Abrus precatorius* (rosary pea), is mainly known as a potent inhibitor of protein synthesis but, in low doses, has also been implicated as triggering autoimmune demyelinating disease, a condition that causes damage to the myelin sheath covering of nerve fibers [1]. This damage to the myelin sheath reduces its insulation ability, consequently interrupting the nerve electrical signals that are necessary for proper muscle function. Sahoo et al. reported for the first time that abrin induces autoimmune demyelinating disease in humans [2]. This autoimmune disease is similar to Guillain-Barré Syndrome (GBS), in which the immune system attacks myelin, the protective covering that surrounds nerve fibers. The exact cause of GBS is unknown, but it is sometimes triggered after respiratory infections, stomach flu [3] or staphylococcal infections [4]. To better understand abrin’s potential role in human autoimmune disease it is necessary to gain a better understanding of the cellular and molecular mechanisms of the action of the toxin in different cell types. This knowledge will help in the design of targeted therapeutic interventions that will enable clinicians to take proactive measures that fully reverse the damage to the myelin sheath.

It is believed that microbial superantigens such as SEA, SED, and SEE, which are secreted by *Staphylococcus aureus*, and streptococcal pyrogenic exotoxin type C (Spe-C) which is secreted by the human pathogen *Streptococcus pyogenes*, trigger an excessive immune response against self-antigens causing chronic inflammation and tissue damage, and are associated with various diseases including autoimmune disorders of the nervous system, rheumatoid arthritis, atopic dermatitis, and Kawasaki disease. It is not yet known what triggers those diseases and there is no agreement over whether those diseases are infectious diseases or immune-mediated diseases [5]. The association between autoimmune diseases, those bacterial species, and their secreted superantigens has been extensively reported. For instance, *Streptococcus pyogenes*, which produces the superantigen Spe-C, was isolated from patients with the autoimmune disease acute rheumatic fever (ARF) [6] and isolated from patients with acute Kawasaki disease [7,8]. It has been shown that high titers of antibodies against SEA were found in sera from patients with atopic dermatitis and Kawasaki disease [9,10]. In addition, strains of *S. aureus* that were isolated from atopic dermatitis patients were found to produce SEA [11,12,13], and high titers of anti-SEA antibody have been documented in several Atopic dermatitis patients [11,14,15,16]. SEA was found to be present in blood samples and synovial fluids from patients suffering from rheumatoid arthritis [17]. It was shown that myelin basic protein (MBP), which is found in the sera of healthy people [18], when injected with low doses of SEA, was able to induce encephalitis in mice that had been previously immunized with MBP but did not show clinical signs of disease [19]. Superantigens trigger excessive production of cytokines, and it is believed that elevated levels of cytokines and an overactive immune response during a cytokine storm can lead the immune system to become unable to distinguish between self and foreign antigens, thus causing the immune system to mistakenly attack itself and damage organs [20,21]. For instance, Liu et al. [22] demonstrated that IFN-γ mediates the development of the autoimmune disease systemic lupus erythematosus (lupus) in humans and Lauwerys and Houssiau demonstrated that IFN-γ increases lupus disease severity in animal models [23].

It is known that the abrin B chain is a lectin with specific binding affinity to the galactose residues on the cell surface membrane [24] and mediates entry of the A:B holotoxin complex into the cell cytosol in a galactose-specific process [25]. In the cytosol, the abrin A chain inactivates ribosomes by cleaving the N-glycosidic bond of adenine at nucleotide position 4324 in the 28s rRNA of the 60s ribosomal subunit [26]. It has been shown that recent activation is necessary for the increased expression of macrophage galactose-type lectin-binding glycans on the cell surface of T-cells [27]. Superantigens initiate CD4^+^ T-cell activation through transcription factors including the nuclear factor of activated T-cells (NFAT) proteins that are regulated by calcium signaling. This subsequently triggers gene expression resulting in T-cell activation, proliferation and the production of cytokines. The objective of the present study is to examine and compare the effect of abrin on superantigen-activated CD4^+^ T-cells and different cell types such as kidney cells, cell surface interactions and the ability of CD4^+^ T-cells to produce cytokines. Milk consists of nearly 1% galactose and is used as a competitive analog of cell surface galactose-bearing glycans to study interaction with abrin. A range of superantigens, that unlike conventional antigens can stimulate large populations of T-cells, are used to activate CD4^+^ T-cells immune cells to study pre-activation on the effects of abrin on those cells. It is shown that abrin toxin induces signal activation that amplifies superantigen activity and boosts, in a dose-dependent manner, cytokine secretion in stimulated T-cells. The ability of abrin to boost the levels of cytokines released may be the mechanism by which abrin toxin triggers autoimmune disease.

## 2. Materials and Methods

### 2.1. Toxins

Abrin toxin, Staphylococcal enterotoxins type A (SEA), SED, SEE, and SPE-C were purchased from Toxin Technology (Sarasota, FL, USA). Toxin purity of 95% or greater was confirmed by SDS-PAGE analysis with the application of Coomassie blue stain. Toxins were prepared in aqueous solution to the concentrations specified. Abrin toxin in PBS retains it activity at 63 °C for 30 min at concentrations greater than 1 ng/mL but was fully inactivated at concentrations lower than 0.1 ng/mL. Storage at temperatures of −20 °C or below, can prolong the shelf life of diluted abrin.

### 2.2. Media and Reagents

Supplemented cell culture medium was comprised of RPMI1640 (Gibco/Thermo Fisher, Waltham, MA, USA) with 10% fetal bovine serum (HyClone, Logan, UT, USA), 1× MEM nonessential amino acids (Gibco) and 100 nM sodium pyruvate (Gibco) supplements. Additional supplements with antibiotics and antimycotics—200 μg/mL hygromycin B (Invitrogen, #10687-010) or 100 units/mL penicillin (Gibco/Thermo Fisher) and 100 μg/mL streptomycin (Gibco/Thermo Fisher) were added as indicated.

### 2.3. Cells and Cell Lines

Vero cells, a commonly used mammalian cell line originating from epithelial cells from an African green monkey kidney, were obtained from the American Type Culture Collection (ATCC, Rockville, MD, USA). Also obtained from the ATCC were CCRF-CEM cells (ATCC CCL-119), TCR Vβ9 expressing T lymphoblasts isolated from a young female Caucasian with acute lymphoblastoid leukemia, and Raji B-cells (ATCC CCL-86), a human cell line established from Burkitt’s lymphoma. A genetically engineered Jurkat (CD4^+^ T-cell leukemia) cell line expressing the luciferase reporter under control of an NFAT response element and the corresponding Bio-Glo luciferase reagent were obtained from Promega (Sunnyvale, CA, USA). Jurkat reporter cells were maintained in supplemented cell culture medium with the addition of 200 μg/mL Hygromycin B. CCRF-CEM and Raji cells were maintained in supplemented cell culture medium with the addition of penicillin and streptomycin. A 37 °C incubator with a humidified atmosphere of 5% CO_2_ was used for the maintenance of all cell lines. The enumeration of viable cells was conducted by trypan blue exclusion and visual counting using a hemacytometer.

### 2.4. Measuring Fluorescence of GFP-Transduced Vero Cells

Generation of the adenoviral-expressed GFP gene and development of the fluorescent Ad-GFP assay was previously described [28,29]. The GFP assay was performed as follows. Vero cells were transferred to 96-well black cell culture plates, part #655090 (Greiner, Monroe, NC, USA), at a concentration of 3 × 10^4^ cells per well in 100 μL of medium. The plates were subjected to an overnight incubation at 37 °C in a 5% CO_2_ incubator to facilitate attachment of the Vero cells to the plate. After 24 h had passed, abrin toxin solution was added to each well to a final concentration in a range from 1 pg/mL to 100 ng/mL. Subsequently, the cells treated with abrin were transduced with GFP-expressing adenovirus at a MOI of 100. GFP fluorescence was measured at 24 h or 48 h post-transduction to quantify the inhibition of GFP production. Prior to fluorescence analysis, the media were removed from the cell culture plates and the cells were washed to minimize autofluorescence caused by media components and to maximize the signal-to-noise ratio. A Synergy HT Multi-Detection Microplate Reader (BioTek, Winooki, VT, USA) was used to analyze the plates with 485/20 nm excitation and fluorescence measured at 528/20 nm.

### 2.5. Measurement of IL-2 Secretion by ELISA

Either CCRF-CEM cells or the Jurkat reporter cell line at a concentration of 2 × 10^6^ cells per mL were transferred to clear 96-well cell culture plates in aliquots of 50 µL per well. To these wells were added an additional 25 µL aliquot of Raji cells at a concentration 2 × 10^6^ cells per mL. Finally, a 25 µL aliquot of SEA, SED, SEE or SPE-C solution was added to each well to achieve the desired final concentration of toxin. The cell culture plates were incubated at 37 °C and supernatant samples withdrawn after 24 and 48 h. The level of IL-2 secreted into the supernatant was measured by ELISA following the protocol provided by the manufacturer (BD OptEIA Human IL-2 ELISA).

### 2.6. Statistical Analysis

Statistical analysis of data was performed using SigmaStat 3.5 for Windows (Systat Software, San Jose, CA, USA). Statistically significant abrin toxin-induced INFγ and IL-2 secretion by CD4^+^ T-cells was determined by applying one-way analysis of variance. Experiments were performed in triplicate and a value of *p* < 0.05 was considered to indicate results that were statistically significantly different. To determine statistically significant difference between treatments and the control, *t*-test analysis was employed.

## 3. Results

### 3.1. Milk Reduces Biological Activity of Abrin to Inhibit Protein Synthesis

**Figure 1:**
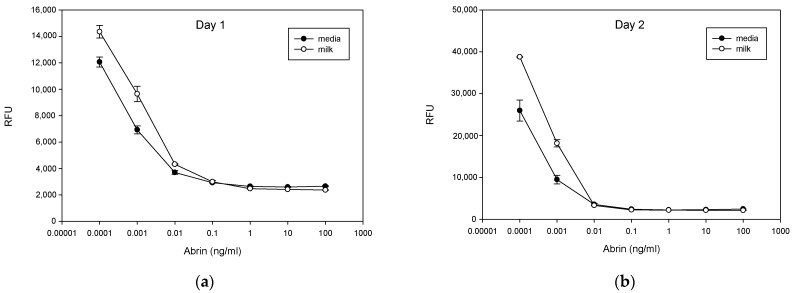
Milk reduces abrin biological activity to inhibit protein synthesis. Transduced Vero cells that were treated with various concentrations of abrin in milk or with cell culture media for 24 h (**a**) and 48 h (**b**), were used to quantitatively determine abrin biological activity. The fluorescence light signals were detected by a plate reader. Error bars represent standard errors.

It has been shown that the abrin toxin B chain which has high binding affinity to the membrane galactose cell-surface receptors mediates the entry of the A:B holotoxin complex into the cytosol, inactivating ribosomes, resulting in the inhibition of protein synthesis. Milk consists of nearly 1% galactose. The ability of milk to competitively bind abrin and reduce the ability of the B chain to bind galactose on the cell surface was studied in an experiment comparing the effect of abrin on the expression of GFP in transduced Vero cells, a commonly used cell line. Milk or control media was spiked with increasing concentrations of abrin toxin (1 pg/mL to 100 ng/mL) and added to GFP-transduced Vero cell seeded 96-well microtiter plates. After 24 h or 48 h of incubation, cells were washed, and fluorescence emission intensity was measured. The measurement of the relative inhibition of the production of green fluorescence protein, as measured by light intensity, is a measure of the ability of abrin to bind and enter the cells. The results (Figure 1) show that there is a strong negative relationship over three logs between abrin concentration and GFP expression: The level of GFP expression was reduced in a dose-dependent manner after treatment with abrin toxin for 24 or 48 h at concentrations of 0.1 pg/mL, 1 pg/mL and 10 pg/mL. Above 10 pg/mL abrin toxin concentration, GFP inhibition reached a plateau. The results also show that the presence of milk reduced the effect of abrin toxin, as indicated by inhibition of the green fluorescent protein synthesis in both tested days. These results suggest that galactose competes for the binding sites on abrin toxin and reduces the number of abrin molecules able to enter the cells.

### 3.2. Abrin Toxin Induces Excessive IL-2 Secretion in SEA-Stimulated Human T-Cell Line CCRF-CEM

**Figure 2:**
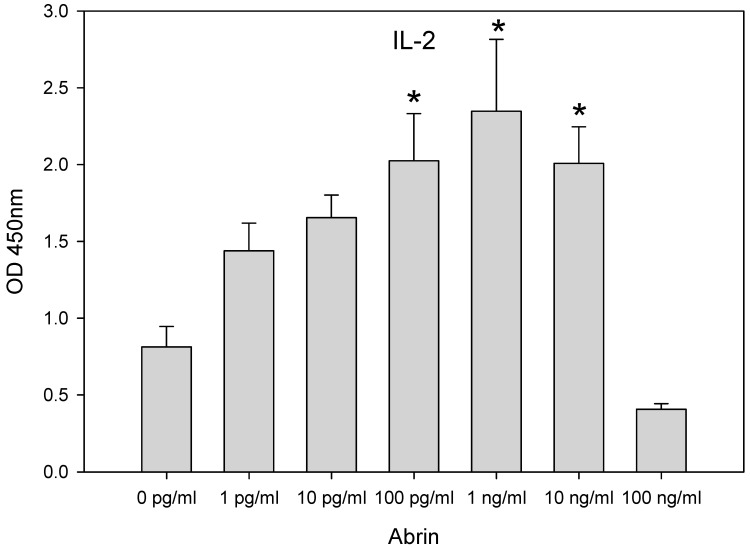
Low doses of abrin induce excess IL-2 secretion in SEA-stimulated T-cells. A mixed culture of Raji B-cells and the human T-cell line CCRF-CEM was co-incubated for 2 h with SEA on a 96-well plate. Abrin toxin was then added to the cells in concentrations ranging from 1 pg/mL to 100 ng/mL with further incubation for 24 h. IL-2 secretion was detected by ELISA. Error bars represent standard errors, and an asterisk indicates significant differences from control.

The observations in Figure 1, as measured by GFP expression, show that abrin toxin, which has binding affinity to galactose, reduces ribosomal activity and protein synthesis in kidney cells at low abrin concentrations and that milk, which contains galactose, interferes with this effect. Galactose is one of the precursors used to build glycans. It was shown by others that Macrophage Galactose-type Lectin (MGL)-binding glycans are expressed in high levels on the cell surface of activated T-cells [27]. Abrin, like MGL, is a galactose lectin and high expression of such glycans may offer more sites for the binding of abrin in the same way that abrin binds to milk galactose. Here, CD4^+^ T-cells following superantigen stimulation were used to investigate the mechanism of action in which abrin toxin triggers effects associated with autoimmune disease. SEA was selected because it had been shown to be able to induce encephalitis in mice that did not show clinical signs of disease after being immunized with MBP. Human T-cell line CCRF-CEM was selected because it expresses the TCR Vβ9 that is responsible for recognizing SEA. The Raji B-cell line was selected to perform the role of an antigen-presenting cell (APC) to present SEA bound to MHC class II to the T-cell receptor Vβ9. A suspension of those two cell types was treated with 100 ng/mL of SEA to stimulate the T-cells. After 2 h of incubation, increasing amounts of abrin toxin were added (1 pg/mL to 100 ng/m), and the cells were further incubated for 24 h. Interleukin-2 (IL-2) secretion was then measured, and the results (Figure 2) show exactly the opposite of what would be expected. Cell ribosomal activity, to provide the large amount of newly synthesized protein needed to support cytokine secretion over a short period of time, apparently increased with the presence of abrin in low to moderate doses. Figure 2 shows for the first time that abrin toxin amplifies the superantigen SEA activity in stimulated CD4^+^ T-cells. Abrin toxin caused IL-2 secretion and there was a dose-dependent correlation between abrin toxin concentration and IL-2 secretion over a 4-log concentration range from 1 pg/mL to 1 ng/mL. Peak secretion of IL-2 occurred at an abrin concentration of 1 ng/mL, but at a concentration of 100 ng/mL, abrin-induced IL-2 secretion diminished. This is not surprising because it shows what would be expected from a potent inhibitor of protein synthesis that is affected via abrin A chain cleavage of the 28s rRNA of the 60s ribosomal subunit.

### 3.3. Abrin Toxin Induces NFAT Signaling Pathway Activation and IFN-γ and IL-2 Secretion in SEE, SED, or SPE-C-Stimulated CD4^+^ T-Cells Expressing TCR Vβ8

**Figure 3:**
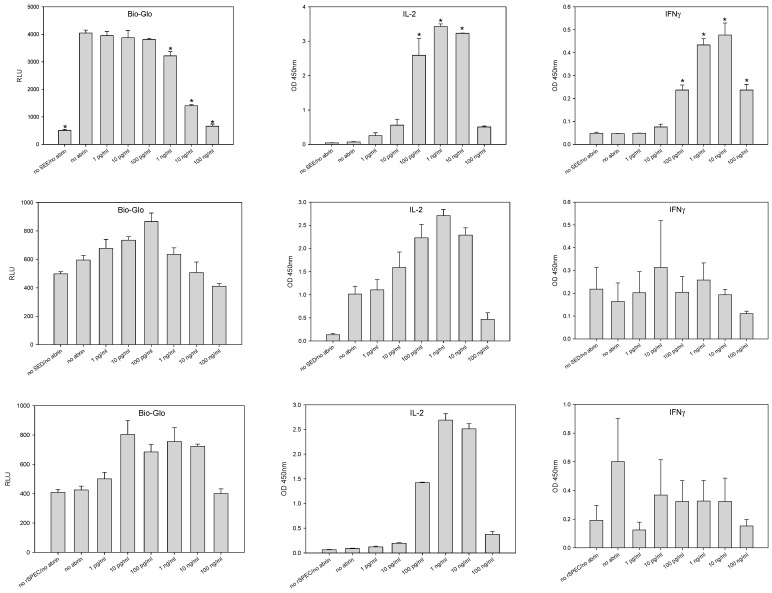
Effect of abrin on NFAT activation and cytokine secretion of T-cells stimulated by different superantigens. Raji B-cells were plated together with engineered Jurkat T-cells (containing a firefly luciferase gene driven by an NFAT response element) in a 96-well plate. The mixed cultures were co-incubated for 2 h with SEE (**top** panels), SED (**center** panels) or SPE-C (**bottom** panels), then various concentrations of abrin toxin ranging from 1 pg/mL to 100 ng/mL were added and further incubated for 24 h. The light emission, IL-2 and IFN-γ secretion were detected by a plate reader. Error bars represent standard errors, and an asterisk indicates significant differences with controls.

The ability of abrin toxin to boost production and release cytokines was next examined using a different CD4^+^ T-cell line with a different Vβ expression than the Vβ9 expressed on the human leukemia CCRF-CEM cell line after stimulation with other microbial superantigens, than SEA that have also been associated with autoimmune diseases. To further understand the molecular mechanism, a genetically engineered Jurkat T-cell-line was used that expresses TCR Vβ8 and the luciferase reporter gene under the regulation of the nuclear factor of activated T-cells response element (NFAT-RE). The Raji B-cell line was utilized as APC. T-cell stimulation was carried out by exposure to staphylococcal enterotoxin type E (SEE) at a concentration of 100 pg/mL, staphylococcal enterotoxin type D (SED) at a concentration of 100 ng/mL or streptococcal pyrogenic exotoxin type C (SPE-C) at a concentration of 1 µg/mL. After a 2 h incubation of the cell mixture with SEE, SED, or SPE-C, abrin toxin was added in concentrations ranging from 1 pg/mL to 100 ng/mL followed by further incubation for 24 h, after which IL-2 and IFN-γ secretion was tested by ELISA and NFAT expression was examined using the luciferase reporter. The results in Figure 3 show that abrin toxin induced activation of the NFAT pathway that coordinates immune response genes and initiated dose-dependent IL-2 secretion by CD4^+^ T-cells that were pre-stimulated with the superantigens SED and SPE-C. The results also show that when the mixed cell culture was treated with SEE, which has a very high affinity to TCR V-β8, it was oversaturated at a concentration of 100 pg/mL of SEE. It reached a plateau to 4000 RLU where there was no further increase in NFAT activation, even after abrin toxin was added. But it induced dose-dependent IL-2 and IFN-γ secretion. At higher doses, abrin’s inhibitory effect on protein synthesis may override the initial increase in activity.

## 4. Discussion

The present study shows that abrin toxin affects protein synthesis differently in different cell types. In the GFP-transduced kidney cell line (Vero cells) there was a strong negative correlation between abrin concentration and GFP expression. It was also shown that milk reduces the effect of abrin toxin as measured by GFP expression. This observation can be explained by competitive binding of the galactose-specific abrin B chain lectin to galactose in milk and the galactose-terminated glycoproteins on Vero cells. Such competition would lead to a reduction of abrin A chain entering the cell cytosol to cleave the N-glycosidic bond of adenine at nucleotide position 4324 in the 28s rRNA of the 60s ribosomal subunit [26]. This is demonstrated by reduced inhibition of the synthesis of green fluorescence protein as measured by fluorescent light intensity emitted by transduced Vero cells. However, abrin toxin was observed to produce different effects in human CD4^+^ T-cells. In these immune cells abrin toxin promotes ribosomal activity. These cell types interact differently to abrin, possibly because they bear different patterns of galactose-bearing ligands on their cell surfaces and consequently affect ribosomal function in a different way. Instead of decreasing the activity of cell ribosomal machinery and inhibiting protein synthesis as abrin did in Vero cells, the toxin does the exact opposite in the immune cells tested. In those cells abrin appeared to promote ribosomal activity, as demonstrated by the secretion of cytokines in pre-stimulated T-cells, where production of newly synthesized proteins would be necessary. The results in this study show that abrin toxin upregulated the intensity of the immune response (as measured by secretion of cytokines) in antigen stimulated CD4^+^ T-cells, in a dose-dependent manner. The excessive secretion of cytokines creates excess inflammation that breaks down immune tolerance and consequently the immune cells may mistakenly attack healthy cells and cause major alterations in tissues, and this may be the causative mechanism in abrin-associated autoimmune demyelinating disease. It is crucial to acknowledge the generalizability of results obtained using human CD4^+^ T-cell lines such as CCRF-CEM cell expressed TCR Vβ9, Jurkat T-cell expressed TCR Vβ8 and Raji B-cell line that perform the role of an antigen-presenting cell, do not completely replicate the complex cellular environment of the diverse population of leukocytes, which can limit the translation of our findings to whole human body physiology.

Previous studies have shown that Macrophage Galactose-type Lectin (MGL)-binding glycans are highly expressed on the cell surface membrane of activated T-cells post-infection. Overnight stimulation of CD4^+^ T-cells with anti-CD3/CD28 or with protein kinase C (PKC) activator, phorbol 12-myristate 13-acetate (PMA), resulted in a robust increase in the expression of MGL-binding epitopes from 3.9% in cell culture media to 32% with anti-CD3/CD28 and 59% with PMA [27]. The MGL-binding galactose-bearing ligands that are highly expressed on stimulated T-cells offer more sites to affect the binding of abrin in the same way that abrin binds to milk galactose. It is proposed that the observation of Ramnath et al., who showed that when abrin toxin was delivered to mice in sub-lethal doses it decreased solid tumor cell numbers in vivo and significantly increased mice survival time [30], which could be explained by abrin toxin amplification of activation of T-cells arriving at the tumor site. It has been proposed that the excessive production of cytokines causes the immune system to mistakenly attack self-antigens and damage organs [20,21]. For instance, Liu et al. and Li et al. [22,31] demonstrated that IFN-γ mediates the development of the autoimmune disease systemic lupus erythematosus (lupus) in humans, and IFN-γ increases lupus disease severity in animal models [23]. Some microbial agents may act in a similar manner to the plant protein abrin that binds galactose-bearing cell-surface glycans on activated T-cells post-infection and induces signal activation in stimulated T-cells that leads to excessive cytokine secretion. The mechanisms responsible for autoimmune disorders that are triggered post-infection, e.g., rheumatoid arthritis, atopic dermatitis and Kawasaki disease that affects the nervous system, may share similarities to the mechanism by which abrin toxin leads to excessive production of cytokines. Likewise, the ability of low doses of SEA to induce encephalitis when co-injected with MBP in mice previously immunized with MBP and showing no sign of disease may share a similar mechanism [19].

It has been shown that myelin basic protein, which plays a critical role in the organization of myelin sheaths in the nervous system, is found in the sera of healthy people [18]. Future work might explore whether the in vitro effects of abrin can be replicated in animal models. This might entail immunizing mice with myelin basic protein followed by induction of demyelinating disease by oral gavage with Staphylococcal enterotoxin type A and with increasing concentrations of abrin toxin. The progress of pathogenesis would then be studied in conjunction with the monitoring of the levels of cytokines in circulating blood, perhaps by the use of cytometric bead array or ELISA directed at specific targeted cytokines.

## Data Availability

All relevant data are within the manuscript.
